# Supplementary material for: Associations of Ultra-Processed Food Intake with the Incidence of Cardiometabolic and Mental Health Outcomes Go Beyond Specific Subgroups—The Brazilian Longitudinal Study of Adult Health
Source: Nutrients. 2024 Dec 12;16(24):4291. doi: 10.3390/nu16244291 (PMC11677051; doi:10.3390/nu16244291)
Supplement: Supplementary file 1 [file nutrients-16-04291-s001.zip › nutrients-3337046-supplementary.pdf]

**SUPPLEMENTARY FILE**

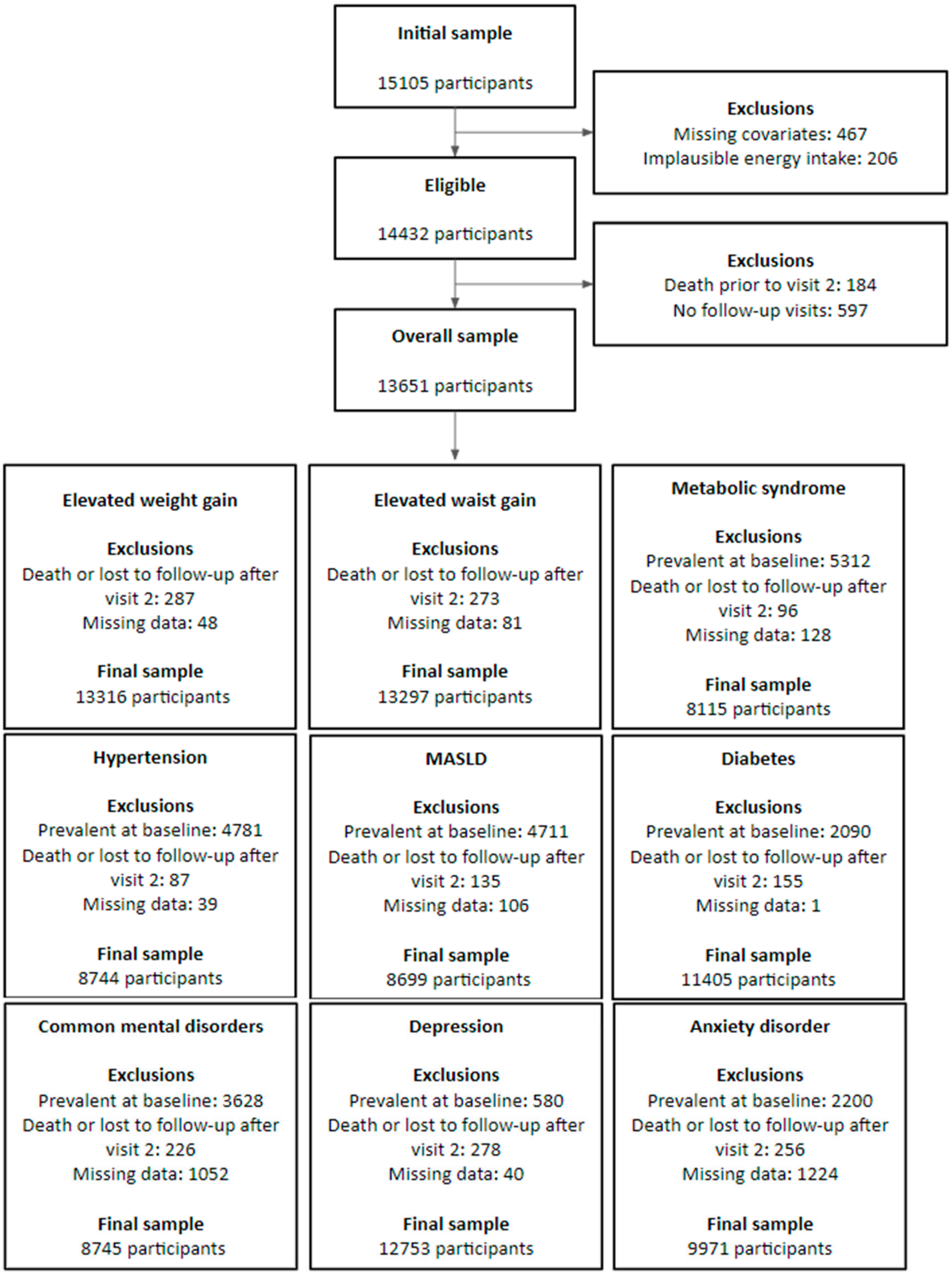


**Supplementary Figure S1**. Flowchart of the analytical sample.

**Supplementary Table S1.** Associations for a one-standard deviation difference in (I) total ultra-processed food (UPF) intake and (II) UPF subgroups, for each outcome.

| | Multivariable model* | Multivariable model + energy intake |
|---|---|---|
| | RR (95%CI) | RR (95%CI) |
| **Elevated weight gain** | | |
| Total ultra-processed food intake | 1.09 (1.04-1.14) | 1.08 (1.02-1.14) |
| Subgroups | | |
| Ready-packaged bread | 1.00 (0.95-1.06) | 1.02 (0.96-1.08) |
| Baked and fried snacks | 1.05 (1.00-1.10) | 1.06 (1.01-1.11) |
| Non-dairy sweet snacks/desserts | 0.99 (0.94-1.05) | 1.01 (0.96-1.07) |
| Spreads | 0.99 (0.94-1.04) | 1.00 (0.94-1.05) |
| Yogurt and dairy sweets | 1.05 (1.00-1.10) | 1.06 (1.01-1.11) |
| Processed Meats | 1.07 (1.02-1.13) | 1.09 (1.03-1.14) |
| Ready-to-eat/heat-mixed dishes | 1.03 (0.98-1.09) | 1.04 (0.99-1.09) |
| Sweetened beverages | 1.02 (0.97-1.07) | 1.03 (0.97-1.08) |
| Distilled alcoholic beverages | 1.09 (1.04-1.15) | 1.09 (1.04-1.15) |
| **Elevated waist gain** | | |
| Total ultra-processed food intake | 1.07 (1.02-1.12) | 1.12 (1.06-1.18) |
| Subgroups | | |
| Ready-packaged bread | 0.99 (0.94-1.05) | 1.02 (0.97-1.08) |
| Baked and fried snacks | 0.98 (0.93-1.04) | 1.01 (0.95-1.07) |
| Non-dairy sweet snacks/desserts | 1.02 (0.97-1.07) | 1.05 (1.00-1.11) |
| Spreads | 0.96 (0.91-1.01) | 0.97 (0.92-1.03) |
| Yogurt and dairy sweets | 1.03 (0.98-1.08) | 1.04 (0.99-1.09) |
| Processed Meats | 1.07 (1.02-1.13) | 1.10 (1.04-1.16) |
| Ready-to-eat/heat-mixed dishes | 1.00 (0.95-1.05) | 1.01 (0.96-1.06) |
| Sweetened beverages | 1.05 (1.00-1.10) | 1.06 (1.01-1.12) |
| Distilled alcoholic beverages | 1.14 (1.08-1.20) | 1.14 (1.08-1.20) |
| **Metabolic syndrome** | | |
| Total ultra-processed food intake | 1.09 (1.06-1.12) | 1.13 (1.09-1.17) |
| Subgroups | | |
| Ready-packaged bread | 0.94 (0.91-0.98) | 0.95 (0.92-0.99) |
| Baked and fried snacks | 1.01 (0.98-1.05) | 1.02 (0.99-1.06) |
| Non-dairy sweet snacks/desserts | 1.00 (0.96-1.03) | 1.01 (0.98-1.05) |
| Spreads | 1.01 (0.97-1.04) | 1.01 (0.98-1.05) |
| Yogurt and dairy sweets | 1.01 (0.98-1.04) | 1.02 (0.98-1.05) |
| Processed Meats | 1.01 (0.98-1.05) | 1.03 (0.99-1.06) |
| Ready-to-eat/heat-mixed dishes | 1.03 (1.00-1.06) | 1.03 (1.00-1.07) |
| Sweetened beverages | 1.09 (1.06-1.12) | 1.10 (1.07-1.13) |
| Distilled alcoholic beverages | 1.00 (0.97-1.03) | 1.00 (0.97-1.03) |
| **Hypertension** | | |
| Total ultra-processed food intake | 1.06 (1.03-1.10) | 1.09 (1.04-1.14) |
| Subgroups | | |
| Ready-packaged bread | 1.00 (0.96-1.04) | 1.01 (0.97-1.05) |
| Baked and fried snacks | 1.02 (0.98-1.06) | 1.03 (0.99-1.07) |
| Non-dairy sweet snacks/desserts | 0.99 (0.95-1.03) | 1.00 (0.96-1.04) |
| Spreads | 0.98 (0.94-1.02) | 0.98 (0.94-1.03) |
| Yogurt and dairy sweets | 0.99 (0.95-1.03) | 1.00 (0.96-1.04) |

| | | |
|---|---|---|
| Processed Meats | 1.02 (0.98-1.07) | 1.03 (0.99-1.08) |
| Ready-to-eat/heat-mixed dishes | 0.97 (0.93-1.01) | 0.97 (0.93-1.02) |
| Sweetened beverages | 1.08 (1.04-1.12) | 1.08 (1.04-1.13) |
| Distilled alcoholic beverages | 1.01 (0.98-1.05) | 1.01 (0.98-1.05) |
| **MASLD** | | |
| Total ultra-processed food intake | 1.12 (1.08-1.16) | 1.15 (1.10-1.19) |
| Subgroups | | |
| Ready-packaged bread | 0.98 (0.94-1.02) | 1.00 (0.96-1.05) |
| Baked and fried snacks | 1.05 (1.01-1.09) | 1.07 (1.03-1.11) |
| Non-dairy sweet snacks/desserts | 0.98 (0.94-1.03) | 1.01 (0.96-1.05) |
| Spreads | 1.00 (0.96-1.04) | 1.01 (0.97-1.05) |
| Yogurt and dairy sweets | 1.01 (0.96-1.05) | 1.02 (0.97-1.06) |
| Processed Meats | 1.09 (1.05-1.13) | 1.11 (1.07-1.15) |
| Ready-to-eat/heat-mixed dishes | 1.00 (0.96-1.04) | 1.01 (0.97-1.05) |
| Sweetened beverages | 1.09 (1.05-1.12) | 1.10 (1.06-1.14) |
| Distilled alcoholic beverages | 1.01 (0.98-1.05) | 1.01 (0.98-1.05) |
| **Diabetes** | | |
| Total ultra-processed food intake | 1.11 (1.07-1.15) | 1.13 (1.08-1.18) |
| Subgroups | | |
| Ready-packaged bread | 1.00 (0.96-1.05) | 1.00 (0.96-1.05) |
| Baked and fried snacks | 0.98 (0.94-1.02) | 0.98 (0.93-1.02) |
| Non-dairy sweet snacks/desserts | 0.98 (0.94-1.03) | 0.98 (0.94-1.03) |
| Spreads | 1.00 (0.96-1.05) | 1.00 (0.96-1.05) |
| Yogurt and dairy sweets | 0.94 (0.90-0.99) | 0.94 (0.90-0.99) |
| Processed Meats | 1.07 (1.03-1.12) | 1.08 (1.03-1.12) |
| Ready-to-eat/heat-mixed dishes | 0.99 (0.95-1.04) | 0.99 (0.95-1.04) |
| Sweetened beverages | 1.14 (1.09-1.18) | 1.14 (1.09-1.18) |
| Distilled alcoholic beverages | 1.03 (1.00-1.08) | 1.03 (1.00-1.08) |
| **Common mental disorders** | | |
| Total ultra-processed food intake | 1.20 (1.15-1.26) | 1.20 (1.14-1.26) |
| Subgroups | | |
| Ready-packaged bread | 1.01 (0.95-1.07) | 1.02 (0.96-1.08) |
| Baked and fried snacks | 1.03 (0.98-1.09) | 1.04 (0.99-1.10) |
| Non-dairy sweet snacks/desserts | 1.08 (1.02-1.13) | 1.09 (1.03-1.15) |
| Spreads | 0.98 (0.93-1.04) | 0.99 (0.94-1.04) |
| Yogurt and dairy sweets | 1.04 (0.98-1.09) | 1.04 (0.99-1.10) |
| Processed Meats | 1.06 (1.00-1.12) | 1.07 (1.01-1.13) |
| Ready-to-eat/heat-mixed dishes | 1.07 (1.01-1.12) | 1.07 (1.02-1.13) |
| Sweetened beverages | 1.10 (1.05-1.16) | 1.11 (1.05-1.17) |
| Distilled alcoholic beverages | 1.03 (0.96-1.10) | 1.03 (0.96-1.10) |
| **Depressive episodes** | | |
| Total ultra-processed food intake | 1.30 (1.20-1.41) | 1.28 (1.16-1.41) |
| Subgroups | | |
| Ready-packaged bread | 1.10 (1.00-1.21) | 1.09 (0.99-1.21) |
| Baked and fried snacks | 1.04 (0.95-1.14) | 1.04 (0.94-1.14) |
| Non-dairy sweet snacks/desserts | 1.13 (1.03-1.22) | 1.12 (1.02-1.23) |
| Spreads | 0.95 (0.86-1.06) | 0.95 (0.85-1.06) |
| Yogurt and dairy sweets | 0.95 (0.86-1.06) | 0.95 (0.86-1.06) |
| Processed Meats | 1.01 (0.91-1.13) | 1.01 (0.90-1.12) |
| Ready-to-eat/heat-mixed dishes | 1.03 (0.93-1.14) | 1.02 (0.93-1.13) |
| Sweetened beverages | 1.23 (1.13-1.34) | 1.23 (1.13-1.33) |

| | | |
|---|---|---|
| Distilled alcoholic beverages | 1.05 (0.92-1.19) | 1.05 (0.92-1.19) |
| **Anxiety disorders** | | |
| Total ultra-processed food intake | 1.15 (1.08-1.22) | 1.11 (1.03-1.19) |
| Subgroups | | |
| Ready-packaged bread | 1.10 (1.03-1.17) | 1.09 (1.02-1.17) |
| Baked and fried snacks | 0.98 (0.91-1.05) | 0.97 (0.90-1.05) |
| Non-dairy sweet snacks/desserts | 1.05 (0.98-1.12) | 1.04 (0.97-1.12) |
| Spreads | 0.99 (0.92-1.05) | 0.98 (0.92-1.05) |
| Yogurt and dairy sweets | 1.02 (0.95-1.09) | 1.02 (0.95-1.09) |
| Processed Meats | 1.08 (1.01-1.15) | 1.08 (1.00-1.15) |
| Ready-to-eat/heat-mixed dishes | 1.05 (0.98-1.12) | 1.05 (0.98-1.12) |
| Sweetened beverages | 1.06 (0.99-1.13) | 1.06 (0.99-1.13) |
| Distilled alcoholic beverages | 1.03 (0.94-1.12) | 1.03 (0.94-1.12) |

*The first column shows the results for the same models presented at Table 2 and 3 of the main manuscript. The multivariable model adjusted in robust Poisson regression includes: age, sex, race/color, school achievement, per capita family income, smoking, physical activity, and alcohol consumption. The second column has an additional adjustment for energy intake.
